# Misplacement of a femoral venous catheter into the ascending lumbar vein: A case report and first use of balloon catheter compression hemostasis

**DOI:** 10.1097/MD.0000000000044472

**Published:** 2025-09-05

**Authors:** Xia Li, Peng Sun, Wanjun Ren, Shizheng Guo, Xiaoping Wang, Qingzhen Gao

**Affiliations:** a Department of Critical Care Medicine, Jinan Shizhong People’s Hospital, Jinan, China; b Department of Nephrology and Blood Purification, Central Hospital Affiliated to Shandong First Medical University, Jinan, China.

**Keywords:** ascending lumbar vein, balloon angioplasty, complication, femoral venous catheter, hemorrhagic shock

## Abstract

**Rationale:**

This case report aims to highlight a rare but life-threatening complication of femoral venous catheterization and to describe a novel endovascular technique for its management. Non-tunneled femoral catheters provide rapid vascular access for emergency dialysis (e.g., arteriovenous graft [AVG] occlusion, hyperkalemia) but carry risks of vascular injury, potentially causing fatal bleeding. This is the first report of hemorrhagic shock due to ascending lumbar vein rupture from femoral catheter misplacement, successfully managed by balloon compression hemostasis.

**Patient concerns:**

A 48-year-old woman with end-stage renal disease presented with hyperkalemia and an occluded AVG. Following ultrasound-guided femoral vein catheterization, she developed recurrent hypotension during dialysis. Suspected anaphylactic shock or abdominal hemorrhage was refractory to anti-allergic therapy, fluid resuscitation, and arterial embolization.

**Diagnoses:**

Digital subtraction angiography (DSA) revealed the catheter tip had perforated the ascending lumbar vein, causing intraperitoneal hemorrhage. DSA clearly identified the injury site and ruled out other causes, confirming hemorrhagic shock secondary to catheter-induced venous rupture.

**Interventions:**

Immediate balloon catheter compression was applied under fluoroscopic guidance. The balloon was precisely positioned at the venous rupture site and inflated to apply direct pressure, sealing the breach for 15 to 20 minutes to promote endothelial repair. This avoided surgical intervention and was particularly suited to this deep, anatomically complex injury.

**Outcomes:**

Post-compression angiography confirmed complete hemostasis. The patient’s hemodynamics stabilized immediately, with no further episodes of hypotension. Hemoglobin levels remained stable throughout the remainder of the hospitalization (post-procedure Hb: 65 g/L, discharged with Hb: 95 g/L). Subsequent dialysis sessions were successful without recurrent bleeding. The patient was successfully transitioned to long-term dialysis access. At 1-month postdischarge telephone follow-up, the patient reported no complications and had successfully undergone AVG thrombectomy at another facility.

**Lessons:**

This first report demonstrates the efficacy of interventional balloon compression for catheter-related deep vein rupture, offering a novel strategy for rapid hemostasis. Clinicians must be vigilant for vascular injury during non-tunneled catheter placement, especially in complex areas, and utilize DSA promptly for diagnosis. Further research into standardized protocols and long-term outcomes is warranted.

## 1. Introduction

Central venous catheterization is a treatment used for initial and emergency dialysis in dialysis patients. Femoral vein catheterization is associated with complications, including puncture site infection, arterial puncture, deep vein thrombosis, catheter misplacement, and vascular damage. Vascular injury associated with femoral venous catheterization is relatively rare (arterial puncture: 1.62%). The common femoral artery at the inguinal puncture site constitutes the most frequently injured vessel.^[1]^ However, it will not lead to life-threatening complications such as pneumothorax, malignant arrhythmia, hematoma compression, and other life-threatening complications,^[2]^ so femoral vein catheterization is safe and easy, which is a good choice for young doctors. However, there is a lack of understanding of the vascular damage caused by femoral vein catheterization into the ascending lumbar vein, resulting in serious complications. In this case report, we report a case of hemorrhagic shock caused by femoral vein catheterization misplacement into the ascending lumbar vein, which was managed by balloon catheter compression.

## 2. Case presentation

### 2.1. Patient information

A 48-year-old woman with end-stage renal disease secondary to autosomal-dominant polycystic kidney disease and concomitant hypertension presented to our hospital with sudden arteriovenous graft (AVG) occlusion identified during routine pre-dialysis assessment. This was her first AVG thrombotic event. She had been receiving thrice-weekly hemodialysis at an external center for 5 years and had undergone placement of a left forearm AVG 6 months earlier. The most recent dialysis session had been completed without complication 2 days before presentation. Baseline medications included amlodipine 5 mg once daily.

### 2.2. Clinical findings

The patient denied any associated symptoms. Ultrasound confirmed AVG occlusion, and laboratory studies revealed a serum potassium concentration of 7.2 mmol/L, consistent with life-threatening hyperkalemia mandating urgent dialysis.

Due to limited night shift staffing in our department, surgical thrombectomy was not feasible. The junior physician administered 30 g of sodium polystyrene sulfonate orally as a temporizing measure. After obtaining informed consent, a temporary non-tunneled dialysis catheter was inserted into the right femoral vein using the Seldinger technique under real-time ultrasound guidance. The arterial lumen of the catheter demonstrated intact functionality, while significant resistance to aspiration was noted in the venous lumen; however, unimpeded infusion under positive pressure was observed. The on-call physician deemed the catheter clinically functional for immediate use. Owing to the emergency of the procedure, post-placement abdominal radiography was omitted to confirm the catheter position. Hemodialysis was initiated immediately after catheter insertion.

Pre-dialysis vital signs were blood pressure 125/75 mm Hg and heart rate 75 beats per minute. Approximately 2 minutes after dialysis initiation, the patient developed sudden altered consciousness and lower back pain, accompanied by a rapid drop in blood pressure to 70/40 mm Hg. Dialysis was immediately discontinued, and intravenous fluid resuscitation restored hemodynamic stability. Intravenous potassium-lowering agents were subsequently administered.

### 2.3. Diagnostic assessment

Suspecting a type A anaphylactic reaction, the clinician administered 5 mg dexamethasone intravenously and substituted the dialyzer. Despite these measures, the patient again developed acute hypotension during the subsequent dialysis session. Antihistaminic therapy proved ineffective, and a repeat hemoglobin level was markedly lower than the pre-dialysis value (pre-dialysis Hb 100 g/L, post-dialysis 60 g/L), prompting consideration of active hemorrhage.

Contrast-enhanced computed tomography of the abdomen and pelvis revealed intraperitoneal and pelvic hemorrhage, most consistent with ruptured autosomal-dominant polycystic kidneys; concomitant hepatic rupture could not be excluded. The patient received packed red blood cells and intravenous vitamin K1 for hemostatic support. Interventional radiology performed selective hepatic and renal arteriography, which disclosed no overt extravasation; nevertheless, empiric arterial embolization of putative bleeding sites was undertaken.

Following embolization, recurrent rapid hypotension recurred, necessitating circuit modification: blood was withdrawn through the femoral vein catheter and returned via the patient’s native vein. With this configuration, the patient remained hemodynamically stable, implicating the femoral vein catheter itself as the precipitant of the prior hypotensive episodes.

### 2.4. Therapeutic intervention

**Figure 1.**
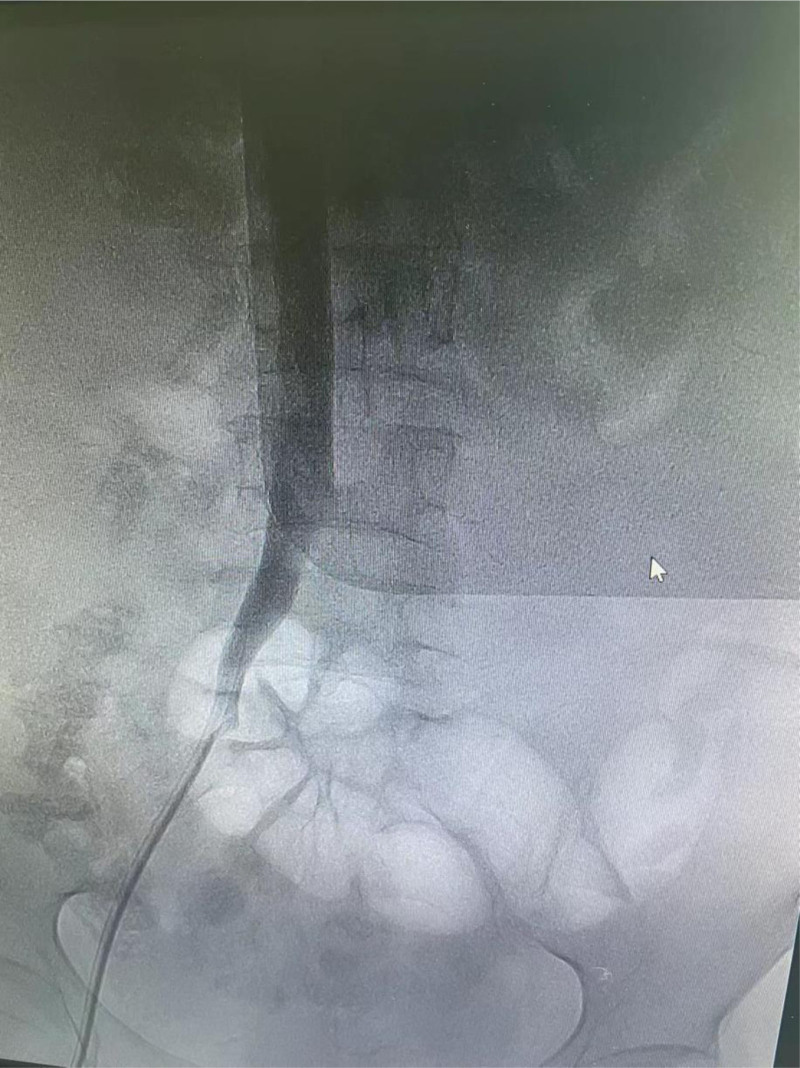
Venography of the right femoral vein catheter showed no abnormality in the arterial end. The contrast agent smoothly entered the inferior vena cava.

**Figure 2.**
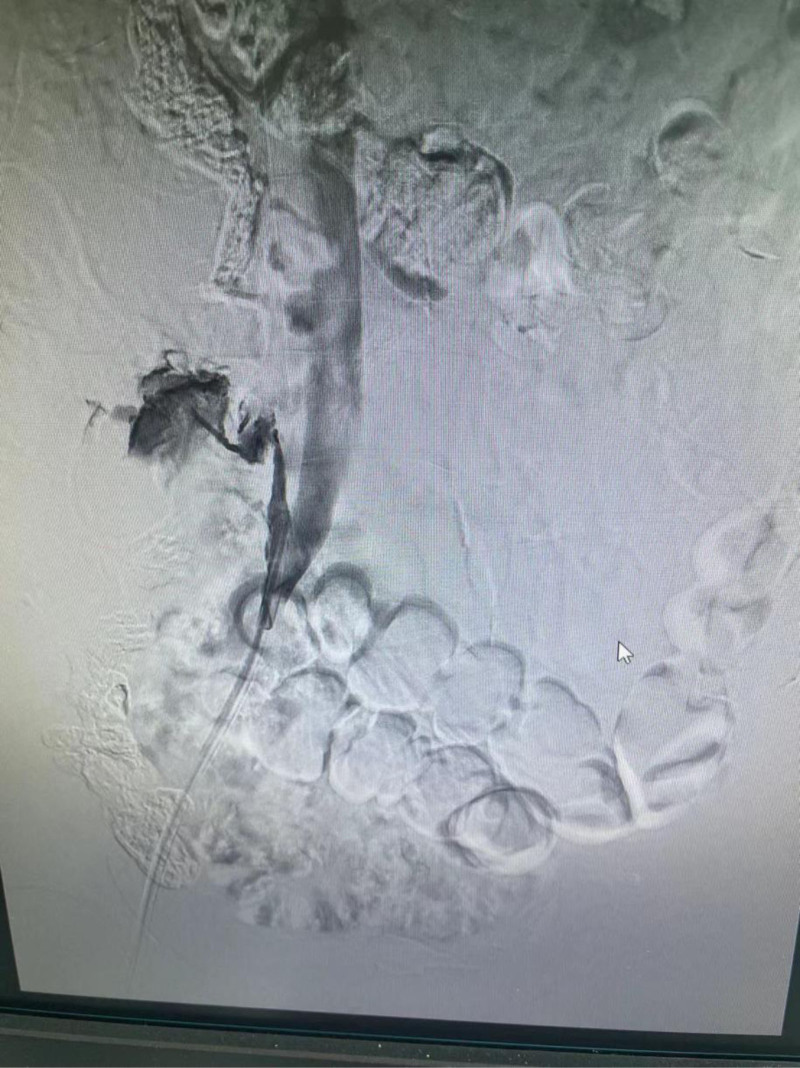
The contrast from the venous end of the right femoral venous catheter showed that the contrast agent had penetrated the abdominal cavity from the right ascending lumbar vein.

Venography via the right femoral venous catheter revealed no abnormalities in the arterial lumen. Contrast medium was successfully delivered into the inferior vena cava (Fig. 1). However, venography through the venous lumen demonstrated active extravasation of contrast from the right ascending lumbar vein into the peritoneal cavity (Fig. 2). We hypothesized that hemorrhagic shock during dialysis resulted from peritoneal hemorrhage secondary to catheter-induced perforation of the ascending lumbar vein, allowing blood reflux from the venous lumen into the peritoneal space.

**Figure 3.**
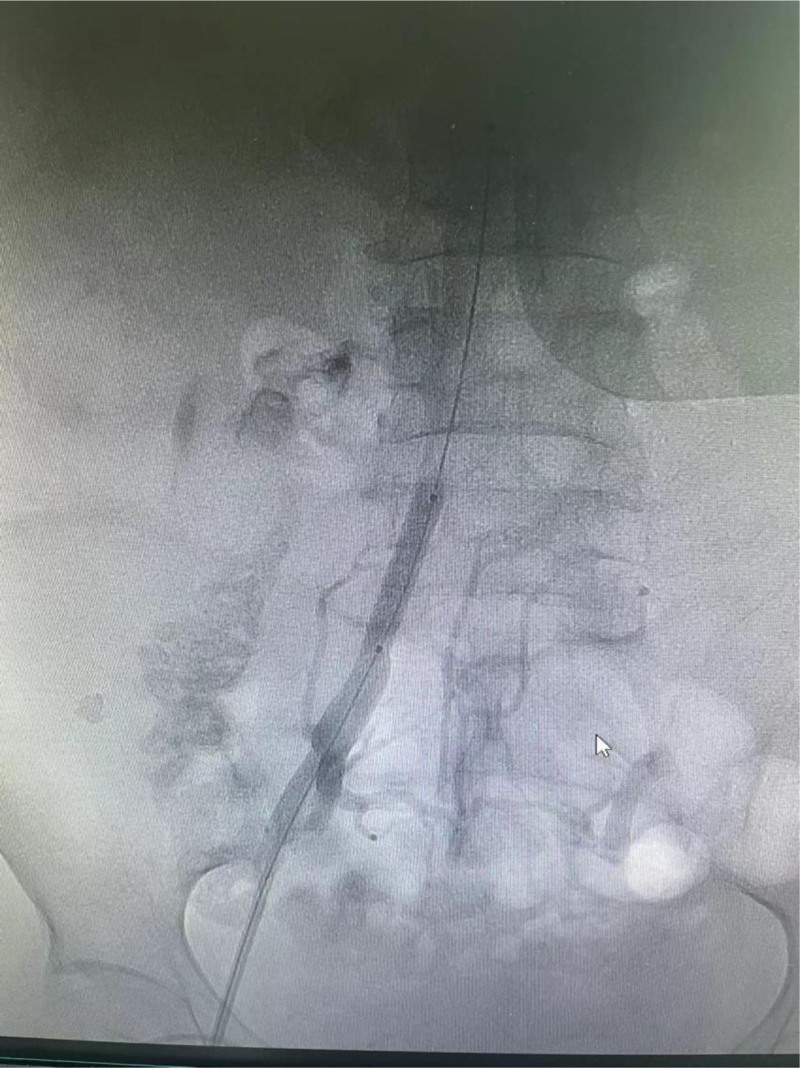
The 8 mm × 40 mm balloon catheter was placed into the opening of the ascending lumbar vein through the guide wire.

**Figure 4.**
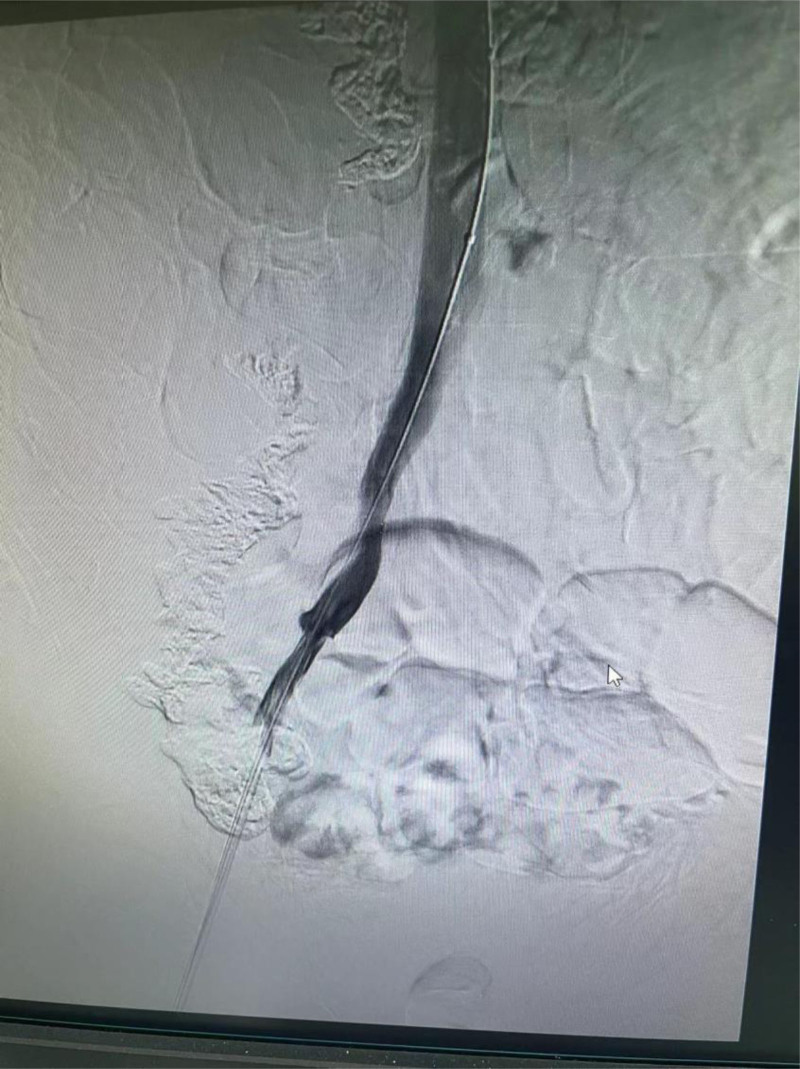
After removing the balloon catheter, angiography again did not show contrast agent leakage.

Surgical intervention was therefore directed at the vascular injury. An exchange guidewire was advanced through the femoral vein catheter into the inferior vena cava, followed by catheter removal and placement of an 8F vascular sheath. A 2.6-m Terumo guidewire was then positioned, over which a Boston Scientific balloon catheter (8 mm × 40 mm) was deployed at the site of perforation (Fig. 3). The balloon was inflated to 2 to 4 atm for 3 minutes per cycle; this maneuver was repeated several times with 5-minute intervals between inflations. Post-procedural venography confirmed complete resolution of contrast extravasation (Fig. 4), indicating successful endovascular repair of the injured vessel.

### 2.5. Follow-up and outcomes

Following the successful endovascular repair, the patient’s vital signs stabilized promptly. Serial hemoglobin measurements showed stability: 65 g/L at 2 hours post-procedure and 80 g/L on the following day. Subsequently, the patient underwent placement of an internal jugular vein catheter for dialysis. The dialysis sessions were well-tolerated without recurrence of hypotension or back pain. Follow-up testing revealed stable hemoglobin levels. During a 1-month post-discharge telephone follow-up, it was confirmed that AVG patency had been restored at another medical facility, and the patient has not exhibited further signs of blood loss.

## 3. Discussion

Femoral vein catheterization is frequently chosen for urgent dialysis access because it is technically straightforward and avoids pneumothorax or arrhythmias associated with central approaches.^[3,4]^ However, femoral vein catheterization carries established risks including catheter-related infection, deep venous thrombosis, and retroperitoneal hematoma. Thrombosis, stenosis, or occlusion of the femoral or iliac veins following catheterization is a well-recognized concern for potential renal transplantation. Damage to these vessels can impair the vascularization of a future transplant kidney. The type and incidence of these complications are related to the disease process, operator’s experience, and operating equipment.^[5]^

Ultrasound guidance for femoral catheter insertion helps obtain an intravenous position of the catheter but may not prevent an eventual malposition on the way up to the vena cava.^[6]^ The femoral vein catheter end is generally in the inferior vena cava.^[7]^ The catheter end position can be determined by a standing abdominal plain film. In the present case, post-procedural abdominal radiography to confirm catheter position was omitted due to urgent clinical circumstances, as transporting the patient to the fluoroscopy suite would have been time-consuming. This deviation from standard practice carried inherent risks and ultimately contributed to the development of hemorrhagic shock. Of course, if the catheter tip mistakenly enters the ascending lumbar vein, it is sometimes not easy to identify through plain film, but ultrasound can diagnose.^[8]^ The right ascending lumbar vein is difficult to detect on anteroposterior abdominal radiographs because it overlaps with the inferior vena cava on anteroposterior radiographs.^[9]^ In this case, the vascular breakage was determined by digital subtraction angiography. However, we do not use fluoroscopy in our routine practice for femoral approaches. However, the standard of care is fluoroscopy for tunneled dialysis catheter insertion, particularly in patients with a history of venous stenosis or thrombosis.^[10]^

The misplacement of central venous catheters is common in newborns and infants, but there are few reports of double-lumen catheter position deviation of the femoral vein in adults.^[11]^ The prominent cases were accidentally entering the left ascending lumbar vein, which is related to the angle between the ascending lumbar vein and iliac vein while entering the right ascending lumbar vein is rarely seen^[12]^; in this case, the catheter perforated the vessel after inadvertent entry, resulting in hemorrhagic shock, which is rarely reported.^[13]^ Zhu et al^[14]^ reported a case in which a central venous catheter was mistakenly placed in the right ascending lumbar vein without symptoms or complications. Catheter misplacement in the ascending lumbar vein may cause neurological symptoms because of its connection to the vertebral venous plexus. Zhang et al^[15]^ reported that complications caused by catheter misplacement in the ascending lumbar vein were observed in >70% of the cases. Additionally, ~20% of the cases involved death due to complications. In the present case, the patient’s complaint of lower back pain suggests catheter position. Previous reports also mentioned low back pain and other manifestations; this uncommon clinical manifestation has great suggestive significance for the diagnosis of abnormal catheter position.

According to previous case reports, most patients were treated by removing the catheter or adjusting the position of the catheter after mistakenly entering the ascending lumbar vein. However, the diameter of the dialysis catheter is 12 Fr, which is thicker than the diameter of the peripherally inserted central catheter in previously reported cases. Due to various reasons, the patient was diagnosed only after the indwelling of catheter for 3 days; directly removing the femoral vein catheter may cause intraperitoneal hemorrhage. Definitive hemostasis was achieved in the interventional radiology suite by balloon catheter compression at the vascular injury site, with subsequent angiography confirming complete resolution of contrast extravasation. Through this case, we reported that the vascular damage was caused by a misplaced femoral vein catheter into the ascending lumbar vein, which resulted in the occurrence of hemorrhagic shock during dialysis. We also reported for the first time a treatment method that can achieve hemostasis by inserting a balloon catheter to compress the damaged site of the blood vessel.

### 3.1. Study limitations

This report has several limitations. First, it is a single-case observation, which inherently limits the generalizability of our findings. The successful outcome achieved with balloon compression hemostasis requires validation in larger cohorts. Second, the definitive diagnosis was delayed due to the initial atypical presentation and the complexity of identifying the exact source of bleeding. This underscores the challenge in promptly recognizing such rare complications. Finally, the long-term integrity of the repaired venous wall remains unknown, as follow-up imaging was not performed due to the patient’s clinical stability and absence of symptoms.

## 4. Conclusion

Dialysis catheter insertion should be performed under ultrasound guidance. The correct position of the catheter tip can be ensured by using digital subtraction angiography. After femoral catheter insertion, if a patient goes into shock during dialysis, the possibility of vascular rupture should be considered, and balloon catheter compression of the vascular damage site is one of the therapeutic options.

## Author contributions

**Methodology:** Shizheng Guo.

**Validation:** Xia Li, Wanjun Ren, Shizheng Guo.

**Writing – original draft:** Peng Sun.

**Writing – review & editing:** Xia Li, Peng Sun, Wanjun Ren, Xiaoping Wang, Qingzhen Gao.
